# The Interaction of Anthracycline Based Quinone-Chelators with Model Lipid Membranes: ^1^H NMR and MD Study

**DOI:** 10.3390/membranes13010061

**Published:** 2023-01-03

**Authors:** Olga Yu. Selyutina, Anna V. Mastova, Nikolay E. Polyakov

**Affiliations:** Institute of Chemical Kinetics and Combustion, Institutskaya St., 3, 630090 Novosibirsk, Russia

**Keywords:** anthraquinones, NMR, molecular dynamics, lipid membranes

## Abstract

Anthracycline antibiotics, e.g., doxorubicin, daunomycin, and other anthraquinones, are an important family of antitumor agents widely used in chemotherapy, which is currently the principal method for treating many malignancies. Thus, development of improved antitumor drugs with enhanced efficacy remains a high priority. Interaction of anthraquinone-based anticancer drugs with cell membranes attracts significant attention due to its importance in the eventual overcoming of multidrug resistance (MDR). The use of drugs able to accumulate in the cell membrane is one of the possible ways of overcoming MDR. In the present work, the aspects of interaction of anthraquinone 2-phenyl-4-(butylamino)naphtho[2,3-h]quinoline-7,12-dione) (Q1) with a model membrane were studied by means of NMR and molecular dynamics simulations. A fundamental shortcoming of anthracycline antibiotics is their high cardiotoxicity caused by reactive oxygen species (ROS). The important feature of Q1 is its ability to chelate transition metal ions responsible for ROS generation in vivo. In the present study, we have shown that Q1 and its chelating complexes penetrated into the lipid membrane and were located in the hydrophobic part of the bilayer near the bilayer surface. The chelate complex formation of Q1 with metal ions increased its penetration ability. In addition, it was found that the interaction of Q1 with lipid molecules could influence lipid mobility in the bilayer. The obtained results have an impact on the understanding of molecular mechanisms of Q1 biological activity.

## 1. Introduction

One of the major problems of chemotherapy is cellular resistance to the drug, which appears after repeated treatments. The multidrug-resistance (MDR) phenotype is frequently associated with a decreased intracellular accumulation of drug that appears to be mediated by a membrane glycoprotein called P-glycoprotein [1,2]. This problem could be overcome by using antitumor drugs which can be concentrated inside cells owing to specific molecular properties [1]. 

**Figure 1:**
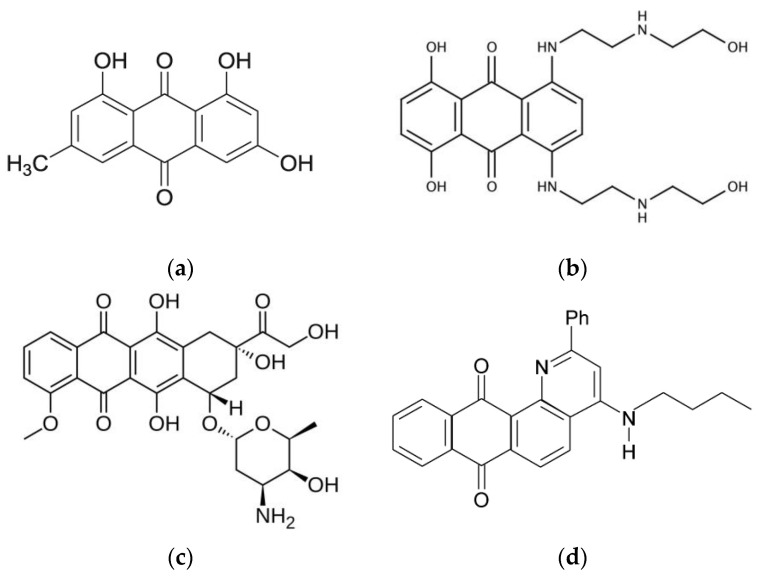
The chemical structures of some anticancer anthraquinones: (**a**) emodin; (**b**) mitoxantrone; (**c**) doxorubicin; and (**d**) anthraquinone Q1 (2-phenyl- 4-(butylamino)naphtho[2,3-h]quinoline-7,12-dione) studied in this work.

Substituted anthraquinones known as anthracycline antibiotics (doxorubicin, daunomycin, emodin, etc.; Figure 1) are widely used in cancer therapy [3]. Two mechanisms are proposed by which these quinones act in cancer cells. The first is the intercalation into DNA duplexes, and the second is generation of reactive oxygen species (ROS) which destroy cellular membranes by stimulation of lipid peroxidation [4,5,6]. Thus, natural anthraquinone derivatives have been shown to be able to induce apoptosis in colon cancer cells by activation of the c-Jun N-terminal kinase pathway via ROS generation [7]. In addition, anthraquinone could cause the loss of mitochondrial membrane potential and changes of the mitochondrial permeability [8]. Another anthraquinone, physcion, was shown to promote both apoptotic and autophagic cell death by modulating transcription factor Sp1 via generation of ROS. Due to the important role of mitochondria in ROS generation and the lipophilic chemical structure of physcion, which allows diffusion of physcion across the mitochondrial membrane, it is supposed that physcion predominantly targets the mitochondria to activate the anti-tumor cascade [9]. Anthracycline are able to influence the cell membrane properties [10]. They could influence glycoprotein synthesis and the transport of small molecules and ions through the cellular membrane and affect the membrane fluidity [11]. In addition, it is suggested that the lipophilic properties of anthraquinones emodin and barbaloin underly their wide biological activity, in particular antimicrobial activity [12]. Several studies have shown that the entry of anthracyclines into tumor cells takes place via passive diffusion [13]. These observations stimulated studies of aspects of drug−membrane interaction for anthraquinones including overcoming MDR [2,14,15]. Additionally, a recent computational study has shown the correlation between anthraquinone activity and its ability to diffuse at a faster rate into bilayers [2].

On the other hand, it is important that especially the damage of the cell membranes as a result of lipid peroxidation is commonly considered as the main mechanism of cardiotoxicity of some anthracycline anticancer compounds widely used in medical practice [16,17]. It was shown, in particular, that doxorubicin could induce ferroptosis in cardiomyocytes [18]. The mechanism of quinone-induced ROS production is shown in Equations (1)–(6):

Q + e → Q^•−^ (1)

Q^•−^ + O_2_ ↔ Q + O_2_^•−^ (2)

(3) $${O_{2}}^{\text{•}-}+{O_{2}}^{\text{•}-}\;\overset{+2H^{+}}{\to }\;H_{2}O_{2}+O_{2}$$

O_2_^•−^ + H_2_O_2_ → O_2_ + HO^−^ + HO^•^ (4)

Q^•−^ + Me^(n)^ → Q + Me^(n−1)^ (5)

H_2_O_2_ + Me^(n−1)^ → HO^−^ + HO^•^ + Me^(n)^ (6)

Quinones mediate the production of ROS by one-electron reduction of a quinone, e.g., by ascorbic acid, by reduced glutathione, or by nicotinamide adenine dinucleotide phosphate (NAD(P)H), yielding the semiquinone radical anion (Equation (1)). Under aerobic conditions, semiquinone radical anions initiate the formation of ROS via the series of reactions shown in Equations (2)–(6) [19,20]. 

The reaction of ROS generation can be accelerated by the presence of trace amounts of transition metal ions such as iron and copper. Copper is an essential trace metal for a number of important biological processes [21]. In particular, the redox cycle between reduced Cu(I) and oxidized Cu(II) states is important for copper catalytic activity as co-factor in enzymes. Copper also contributes to ATP production in mitochondria and ROS detoxification [22]. On the other hand, while copper plays an important role in healthy organisms, enhanced copper levels in tumors lead to cancer progression [21]. Indeed, it has been shown that high copper levels in serum and tissue of cancer patients promote tumor growth and metastasis [23].

Taking into account the effects of copper on tumor growth and progression, chelate-based therapy is intensively developed now as a novel anti-cancer strategy. For example, a class of thiosemicarbazone compounds that effectively bind copper have shown high anti-cancer activity [24]. Thiosemicarbazones have a unique mechanism of action, as they form redox active copper complexes in the lysosomes of cancer cells [24].

Since some anthracycline antibiotics also are able to form metal complexes, they have already demonstrated anticancer activity [17,25]. In addition, metal−anthracycline complexes are able to destroy the cell membrane and DNA via oxidative stress [26,27]. Copper plays an important role in anthraquinone anticancer activity. ROS generation via reduction of Cu(II) to Cu(I) mediated by natural reductants has been assumed as the major mechanism underlying the anticancer activity of copper complexes. Thus, it was shown that doxorubicin induces oxidative DNA damage in the presence of Cu(II) through oxidation of its p-hydroquinone moiety by copper ion [28]. The similar results were obtained for influence of copper on DNA damage by pirarubicin [29]. Another example is the effect of the Cu(II)−mitoxantrone complex on the DNA synthesis of HL-60 human leukemia cells [30]. It was demonstrated that the copper complex shows a stronger ability to inhibit DNA synthesis of the tumor cells than free drug. It is suggested that Cu(II)-mediated oxidative DNA damage may be a common mechanism for the antitumor effects of anthracyclines [28,31]. In addition, Cu(II)−doxorubicin complexes demonstrate lower cardiotoxicity than free doxorubicin [32]. 

In this study, we focused our attention on the anthraquinone that possess anti-cancer activity, namely 2-phenyl-4-(butylamino)naphtho[2,3-h]quinoline-7,12-dione, (Q1; Figure 1) [20,33]. Earlier, it was demonstrated that quinone-chelator Q1 can be effectively reduced by ascorbic acid, glutathione, and NADH with the formation of free semiquinone radical as well as ROS [20,33]. For some cancer cell lines, Q1 showed higher activity in ROS generation than doxorubicin [20]. In addition, it has been shown that Q1 forms chelate complexes with Fe(II) and Cu(II) ions which are redox-active in the linoleic acid micelles oxidation [34]. 

**Figure 2:**
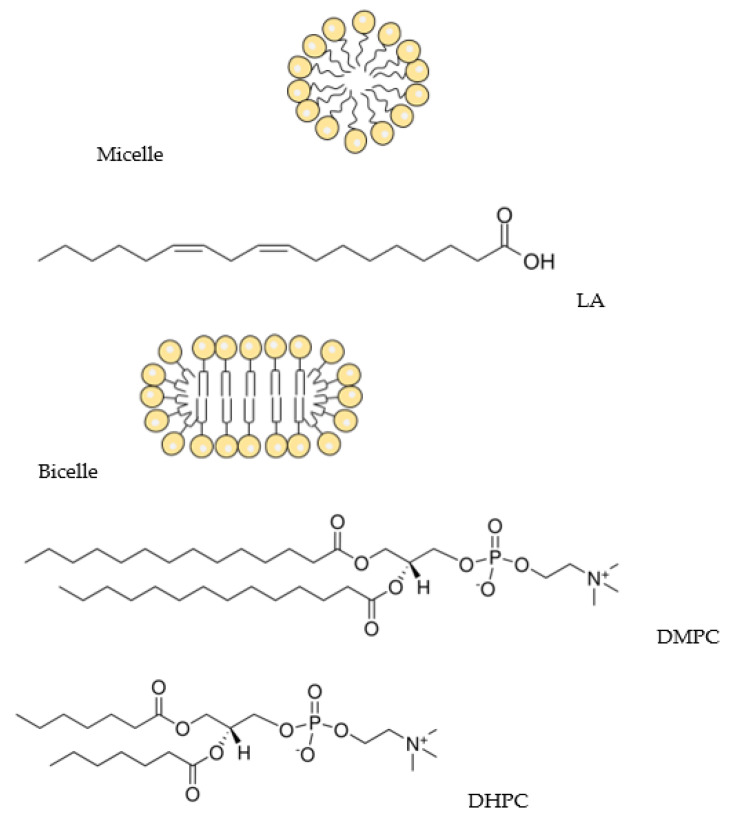
Schematic representation of the micelle and the bicelle and structures of linoleic acid (LA), DMPC (1,2-dimyristoyl-sn-glycero-3-phosphocholine), and DHPC (1,2-diheptanoyl-sn-glycero-3-phosphocholine).

In the present work, the interaction of Q1 and its chelate complex with Cu^2+^ ions with lipid membranes were studied by means of NMR spectroscopy in the model systems, 1,2-dimyristoyl-sn-glycero-3-phosphocholine/1,2-dihexanoyl-sn-glycero-3-phosphocholine bicelles, and linoleic acid micelles (Figure 2) widely used as the models of living cell membranes in the NMR experiments [35,36]. In addition, the molecular dynamics simulation of the Q1 interaction with 1,2-dimyristoyl-sn-glycero-3-phosphocholine bilayer was performed.

## 2. Materials and Methods

### 2.1. Materials

Quinone-chelator, 2-phenyl-4-(butylamino)naphtho[2,3-h]quinoline-7,12-dione (Q1; Figure 1) was synthesized according to the procedure described by Dikalov et al. [37]. Linoleic acid with a >99.0% purity was purchased from Shanghai Aladdin Bio-Chem Technology Co., Ltd., Shanghai, China. A deuterated solvent D_2_O (99.9%D, Sigma Aldrich) were used as received. Bicelles were formed from DMPC (1,2-dimyristoyl-sn-glycero-3-phosphocholine) and DHPC (1,2-diheptanoyl-sn-glycero-3-phosphocholine, purity > 99%; Avanti Polar Lipids; Figure 2). Powdered components (lipids, Q1) were dissolved in chloroform, the solvent was dried, and the resulting film was hydrated with D_2_O. DCl was added to enable the solution to have the pH of 4. To accelerate the formation of bicelles, three freeze-thaw cycles were performed. The DMPC:DHPC molar ratio was 1:2, with the total lipid concentration being 12 mM, Q1 concentration was 1 mM, the Q1−Cu(II) complex concentration was 0.5mM. For experiments in linoleic acid micelles, LA concentration was 12 mM, and samples were prepared in PBS (pH = 7.4). All experiments were conducted at the natural oxygen level.

### 2.2. NMR Study 

^1^H NMR and selective NOESY spectra were recorded on a Bruker Avance HD III NMR spectrometer (500 MHz ^1^H operating frequency). T_1_ relaxation times were measured using a standard inversion-recovery pulse sequence. All experiments were conducted at 303 K.

### 2.3. Molecular Dynamics Simulations

Molecular dynamics simulations were performed to understand the interactions of Q1 with phospholipid-containing membranes using the GROMACS 2018.4 package and GROMOS54a7 force field. The topology of Q1 was built using the Automated Topology Builder [38]. For lipid simulations, the model lipid DMPC (1,2-dimyristoyl-sn-glycero-3-phosphocholine) introduced by Poger and Mark was utilized [39]. The simple point charge (SPC) model of water molecules was used.

The simulation was performed in an NPT ensemble with a constant pressure (1 bar) and a constant temperature T of 303 K, which were maintained by the semi-isotropic Parrinello–Rahman barostat [40] and the Nose–Hoover thermostat [41]. For electrostatic interactions, the PME method with the fourth-order cubic interpolation and a grid of 0.16 was used [42]. The initial configuration of the system contained the bilayer consisting of 128 lipid molecules surrounded by water (~10,000 water molecules) and Q1 molecule located in water outside the bilayer. One production run of a 500 ns duration was performed.

### 2.4. Relative Lipophilicity (log P) Determination

Relative lipophilicity values of Q1 and the Q1−Cu(II) complex were measured using UV-Vis spectroscopy. Q1 and the Q1−Cu(II) complex in concentrations of 0.01mM, 0.02 mM, 0.04 mM were dissolved in deionized water and were stirred for 2 h. Then, the same volume of 1-Octanol was added and samples were stirred for 24 h to reach equilibrium. After that, concentrations of Q1 and the Q1−Cu(II) complex in 1-Octanol were measured using optical absorption spectroscopy. 

## 3. Results and Discussion

### 3.1. Q1 and Q1−Cu(II) Interaction with Linoleic Acid Micells

Interaction of drug molecules with lipid membranes and the exact knowledge of their binding site and distribution in lipid bilayers is of great importance for novel drug development. Many factors influence the interaction of drugs with the cell membrane, such as lipophilicity, size, solubility, and charge [43]. Lipophilicity and charge are key aspects of pharmacopoeia that determine their biological activity [43,44,45]. The measured relative lipophilicity (log P) of Q1 was 1.4 ± 0.1, and log P of the Q1−Cu(II) complex was 1.3 ± 0.1. It is comparable with log P values for doxorubicin (log P = 1.3 [46]), and emodin (log P = 1.74 [47]) and higher than the value for mitoxantrone (log P = 0.79 [48]). Therefore, all mentioned anthraquinones were quite lipophilic, but lipophilicity is not the single factor affecting membrane permeability. Therefore, for another chelator, thiosemicarbazone Dp44mT which is even more lipophilic than anthraquinones (log P = 2.19 [49,50]), it was shown that it cannot penetrate deeply into the lipid bilayer, remaining bound to the surface and staying outside the bilayer for a significant part of the time [47]. For anthraquinone emodin, it was shown that it is bonded to the surface of the lipid bilayer and oriented parallel to it [47,51].

**Figure 3:**
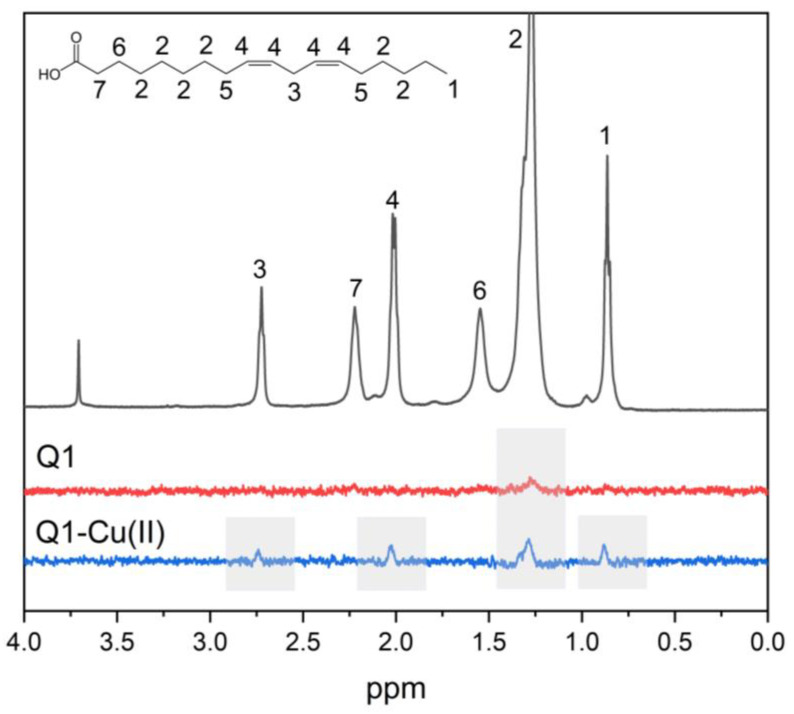
1D NOESY spectra of Q1 (red line) and the Q1−Cu(II) complex (blue line) and ^1^H NMR spectra (gray line) of Q1 in LA micelles. pH = 7.4. Q1 concentration was 1mM.

The ^1^H NMR NOESY technique allows measuring localization and distribution of drug molecules in membranes. The cross-peaks intensities are proportional to the contact probability between corresponding protons and therefore an ideal tool to study intermolecular interactions in membranes. In the present study, we have applied this technique to study the interaction of Q1 and the Q1−Cu(II) complex with linoleic acid micelles. Selective 1D NOESY spectra of Q1 and the Q1−Cu(II) complex in micelles are given in Figure 3. Selective excitation of aromatic Q1 protons was performed.

The cross-peak of the signals of aromatic protons of Q1 with LA signals of (CH_2_) groups was observed. Cross-peaks of NOESY spectra were observed, when the distance between the nuclei was less than 0.5 nm. The obtained result means that Q1 molecule was able to penetrate into LA micelles. It should be noticed that additional cross-peaks appeared for the Q1−Cu(II) complex. This is especially interesting in light of the fact that the values of log P for Q1 and the Q1−Cu(II) complex were practically the same. The appearance of the cross-peaks between Q1 aromatic protons and LA –HC=CH−, =HC−CH_2_−CH=, and −CH_3_− protons means that the Q1−Cu(II) complex was located deeper in the hydrophobic part of the micelle. 

**Figure 4:**
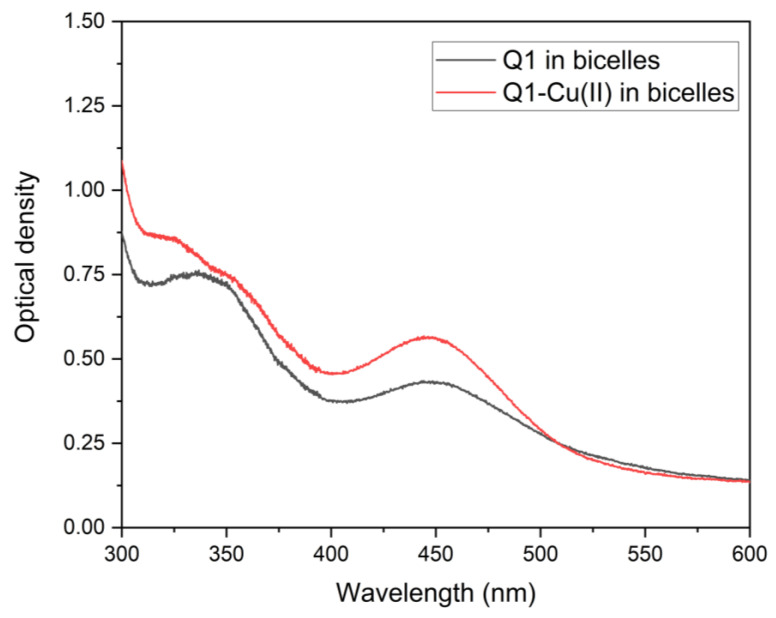
Optical absorption spectra of Q1 and the Q1−Cu(II) complex in LA micelles. pH = 7.4. Q1 concentration was 0.02 mM. CuCl_2_ concentration was 0.01 mM.

To make sure that the Q1−Cu(II) complex was present in this system, additional experiments were performed using optical spectroscopy. The absorption spectra of Q1 and the Q1−Cu(II) mixture in linoleic acid micelles are shown in Figure 4. When copper was added, changes in the spectrum characteristic of Q1−Cu(II) complexes were observed (changes of the optical density at 340 and 450 nm) [34]. The same result was obtained for the Q1−Cu(II) complex in bicelles.

These results could explain the influence of Q1 metal complexes on the rate of LA peroxidation [34]. Q1−Cu(II) complexes are able to penetrate into LA micelles and enhance ROS formation near the hydrophobic “tail” of lipid, where the target of the initiation stage of the lipid peroxidation is placed [52,53,54]. Recall that the quinone-induced peroxidation of LA is initiated by bis-allylic hydrogen abstraction followed by the reaction with molecular oxygen and the formation of peroxyl radicals of lipids [47].

### 3.2. Q1 and Q1−Cu(II) Interaction with DMPC/DHPC Bicelles

**Figure 5:**
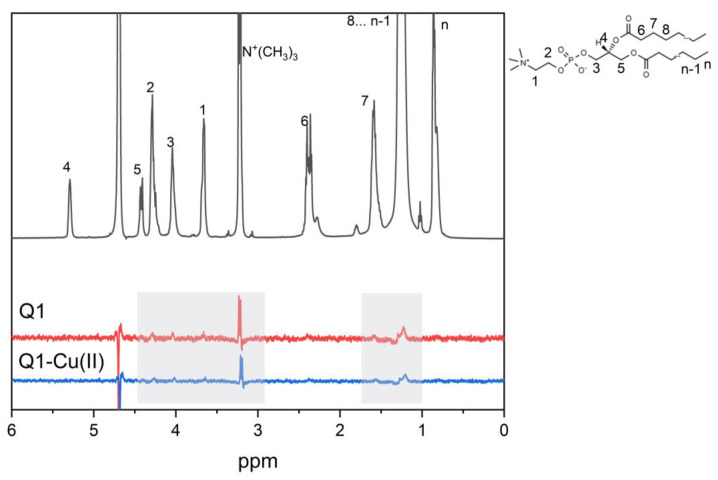
1D NOESY spectra of Q1 (red line) and the Q1−Cu(II) complex (blue line) and ^1^H NMR spectra (gray line) of Q1 in DMPC/DHPC bicelles. pH = 4. Q1 concentration was 1 mM.

At the next stage, experiments were performed at the more relevant membrane model, DMPC/DHPC bicelles. Selective 1D NOESY spectra of Q1 and the Q1−Cu(II) complex in bicelles are shown in Figure 5. Due to the low solubility of Q1 at neutral pH, experiments were performed at pH = 4. DMPC/DHPC bicelles are stable at a pH range of 4−7 [55]. The cross-peaks of the signals of aromatic protons of Q1 with phospholipid signals of acyl (CH_2_) groups, N^+^(CH_3_)_3_ groups and 1, 2, and 3 protons (Figure 5) were observed. It means that same as in the case of LA micelles Q1 penetrated into the lipid bilayer. The absence of the cross-peak with the terminal CH_3_ group means that Q1 did not reach the middle of the bilayer and was located inside the hydrophobic part of the bilayer but near its surface. The same results were observed for Q1 and the Q1−Cu(II) complex.

In addition, spin-lattice (T_1_) relaxation times of lipid protons in the absence and in the presence of Q1 were measured. Nuclear relaxation times T_1_ and T_2_ are very sensitive to molecular mobility and intermolecular interactions. This is why relaxation times could be used to study drug−membrane interactions [56,57]. Spin-lattice relaxation times T_1_ of lipids are determined by high-frequency vibrations of the acyl chain [58,59]. T_1_ relaxation times of lipids in the absence and in the presence of Q1 are given at the Table 1.

It could be seen from Table 1 that the mobility of phospholipid CH_2_ groups were the most affected by the presence of Q1. The mobility of membrane lipids could affect the activity of membrane-associated proteins [60]; therefore, attention should be paid to this aspect of Q1 activity. In addition, the changes of the lipid mobility could be the reason of the membrane permeability changes observed for other anthracycline antibiotics [8].

**Figure 6:**
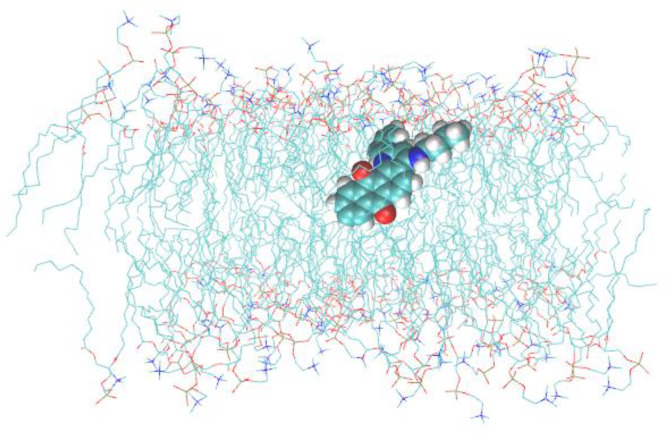
Snapshot of the MD trajectory of Q1 in the box with DMPC bilayer. Water molecules are not shown.

**Figure 7:**
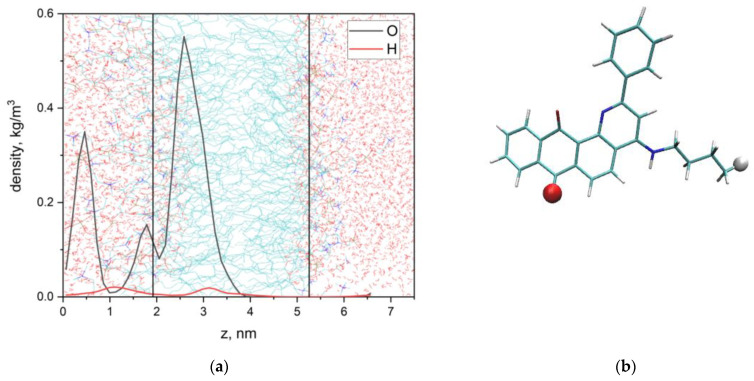
Density profiles of the selected O and H atoms of Q1 (**a**) and atom selections (**b**). Vertical lines correspond to the centers of density profiles of DMPC N-atoms.

These experimental results were confirmed by MD simulations. Figure 6 illustrates the localization of Q1 in the membrane. Q1 molecule quickly (~6 ns) penetrated into the lipid bilayer. Figure 7a shows the calculated density profiles of the selected H and O atoms (see Figure 7b) across the box. The lipid bilayer was centered at the center of the box.

The maximum of density of O-atom was located at 2.6 nm, and the bilayer center was located at 3.7 nm. It means that the aromatic part of Q1 was located near the phospholipid acyl chain and did not contact with terminal CH_3_ groups. Additional maxima at 0.5 nm and 1.8 nm means that the aromatic part of Q1 could leave the hydrophobic part of the bilayer and contact with N^+^(CH_3_)_3_ groups of lipids. These results differed from the data obtained earlier for another anthraquinone, emodin. Emodin could form hydrogen bonds with lipid bilayer surface groups and is located predominantly on the membrane surface [51]. 

**Figure 8:**
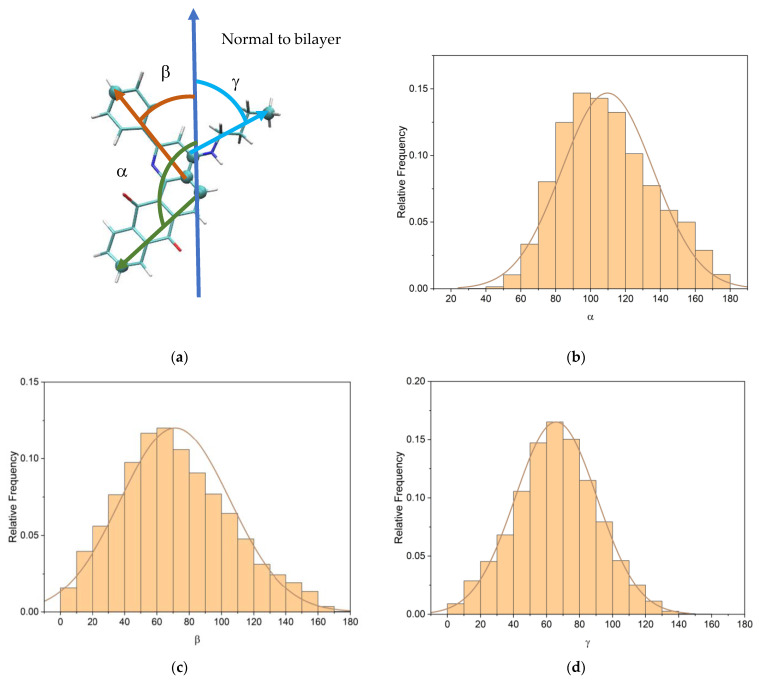
(**a**) Angles selections; (**b**) distribution of the angle between the bilayer normal and the tricyclic part vector of Q1; (**c**) phenyl part vector of Q1; and (**d**) aliphatic part vector of Q1.

Q1 molecule could freely rotate in the bilayer, but the angle between the tricyclic ring (containing quinoid groups) vector and the bilayer normal was predominantly about 100° (Figure 8). The orientation of Q1 in the lipid bilayer is similar to other anthraquinones, which is oriented perpendicular to the bilayer normal [2]. Such an orientation could result in significant changes in lipid packing, which could, in turn, influence lipid mobility.

To estimate the rate of membrane insertion of the anthraquinone, we used the protocol described in [2,61]. The equilibrium constant of the insertion could be obtained from the following equation:

$$D+M\overset{K_{mem}}{↔}\;DM$$

where *D* is the drug, *M* is the membrane, *DM* is the drug bound to the membrane, *K_m_*_em_ is the equilibrium constant of reaction:

$$K_{mem}=\frac{k_{in}}{k_{out}}$$

where *k_in_* is the rate constant of the drug insertion into the membrane, *k_out_* is the rate constant of the drug release from the membrane. The association constant *k_in_* for a ligand in the box, to reach the absorbing surface, is inversely related to the average mean first-passage time for the ligand to hit the surface [2,61]:

$$k_{in}=\frac{AL_{z}}{\langle W\rangle }$$

where *A* is the size of the phospholipid surface, *L_z_* is the thickness of the water layer in which the drug diffuses, and <*W*> is the average mean first-passage time for the drug to hit the surface. *k_in_* could be used as a metric to quantify the membrane insertion propensity of the anthraquinone [2]. The calculated k_in_ for Q1 was 2.6 × 10^9^ s^−1^. It was higher than values obtained for doxorubicin (2 × 10^9^ s^−1^), epirubicin (1.4 × 10^9^ s^−1^), idarubicin (1.5 × 10^9^ s^−1^), and daunorubicin (1.77 × 10^9^ s^−1^) in [2].

## 4. Conclusions

In this study, it was shown that substituted anthraquinone Q1 (2-phenyl-4-(butylamino)naphtho[2,3-h]quinoline-7,12-dione) and Q1−Cu(II) complexes could penetrate into lipid bilayers and linoleic acid micelles in model systems. It was also found that Q1 could influence the lipid mobility. Lipids play an important role in membrane-associated protein folding and functioning [62], and therefore, lipid mobility could influence the properties of membrane-associated proteins. This may contribute both to the activity of anthraquinones and to their side effects. The possibility of the penetration of different anthraquinones into lipid bilayers was studied previously, and it was noticed that the higher success of membrane insertion of anthraquinone correlates with the higher anticancer activity [2,63,64]. Although anticancer activity of anthraquinones is mainly due to direct interaction with nucleic acids, their interaction with cell membranes plays a significant role in its activity [65]. Even if the main mechanism of the drug activity is the interaction with nucleic acids, the drug must pass through a variety of other organelles to reach the DNA. Since anthraquinones are known to cross the cell membrane by passive diffusion, interaction with lipid membranes is an unavoidable step in their activity [13]. In addition, the correlation was found between the cytotoxicity of anthracycline antibiotics and their lipophilicity and ability to penetrate into the lipid bilayer [2]. The obtained results makes an impact on the understanding of molecular mechanisms of Q1 action. The important feature of Q1 is its ability to chelate metal ions, especially transition metal ions, Fe and Cu, responsible for ROS generation in vivo [20,34]. The present study demonstrated the increase of penetration ability of Q1 in chelate complexes into linoleic acid micelles. Copper plays an important role in the anticancer activity of anthracycline antibiotics [28,32]. Copper−anthraquinone complex formation could reduce cardiotoxic effects of anthracycline antibiotics, but the mechanism of its effect is unclear. The obtained results allow a better understanding of the molecular mechanism of action of anthracycline antibiotics and their copper complexes.

Clinical use of anthracycline antibiotics is often limited by the appearance of drug resistance of tumors cells during treatment. Moreover, such cell lines become resistant to other drugs [15]. Multidrug resistance is associated with low penetration of drug into the cell. Several studies indicate that this could be overcome by the use of drugs which could accumulate in the cell membrane [14,15]. In addition, different studies show that anthracycline antibiotics demonstrate antitumor activity even in the case when they do not penetrate into the cell and, consequently, do not reach DNA [14,65]. Therefore, the interaction of anthracycline antibiotics with cell membrane and its accumulation in the lipid bilayer plays an important role in its antitumor activity. One of the possible mechanisms of activity in the cell membrane could be ROS generation inside the cell membrane, lipid peroxidation with subsequent damage of the cell membrane. In the previous work, we have demonstrated that Q1−Cu(II) complexes could enhance lipid peroxidation in model systems (linoleic acid micelles) [34]. In the present work, we have demonstrated that the Q1−Cu(II) complex penetrated deeper into the lipophilic environment. Taking into account obtained results, this Q1−Cu(II) complex should be further studied as a promising anticancer agent.

## Figures and Tables

**Table 1:** Spin-lattice (T_1_) relaxation times of lipids in the absence and in the presence of 1 mM Q1.

	N^+^(CH_3_)_3_	CH_2_	CH_3_
*w*/*o* Q1	0.81 ± 0.06 s	1.120 ± 0.04 s	1.370 ± 0.07 s
with Q1	0.7 ± 0.07 s	0.8 ± 0.08 s	1.170 ± 0.1 s

## Data Availability

Data are contained within the article.
